# Trimethylamine N-Oxide in Heart Failure: A Meta-Analysis of Prognostic Value

**DOI:** 10.3389/fcvm.2022.817396

**Published:** 2022-02-16

**Authors:** Xingxing Li, Zongjing Fan, Jie Cui, Dong Li, Jinjin Lu, Xiaoyun Cui, Liandi Xie, Yang Wu, Qian Lin, Yan Li

**Affiliations:** ^1^Second Clinical School of Medicine, Beijing University of Chinese Medicine, Beijing, China; ^2^Department of Cardiology, Dongfang Hospital, Beijing University of Chinese Medicine, Beijing, China; ^3^Department of Cardiology, Dongzhimen Hospital, Beijing University of Chinese Medicine, Beijing, China

**Keywords:** heart failure, trimethylamine N-oxide, major adverse cardiovascular events, all-cause mortality, meta-analysis

## Abstract

**Objective:**

The present study aimed to explore the prognostic value of trimethylamine N-oxide (TMAO) in heart failure (HF).

**Methods:**

PubMed, Excerpta Medica Database (EMBASE), Cochrane Library, Web of Science, Wanfang Database, SINOMED, China Science and Technology Journal Database (VIP), and China National Knowledge Infrastructure (CNKI) were searched up to June 1, 2021. Studies recording the major adverse cardiovascular events (MACEs) or all-cause mortality in HF patients and their circulating TMAO concentrations were included. Meta-analysis was performed using Stata 13.0.

**Results:**

Ten articles (12 studies) involving 13,425 participants from 2014 to 2021 were considered. Compared to low-level TMAO, elevated TMAO was correlated with MACEs and all-cause mortality in HF (RR: 1.28, 95% CI: 1.17, 1.39, *P* < 0.0001, random-effects model and RR: 1.35, 95% CI: 1.28, 1.42, *P* < 0.0001, random-effects model, respectively). Consistent results were obtained in all examined subgroups as well as in the sensitivity analysis.

**Conclusion:**

Elevated TMAO may be an adverse prognostic indicator in patients with HF.

**Systematic Review Registration:**

https://www.crd.york.ac.uk/prospero/display_record.php?RecordID=267208

## Introduction

Heart failure (HF) is an increasing public health problem worldwide (1). Evidence from epidemiological studies has shown that approximately 64.3 million people worldwide suffer from HF (2). Patients with HF usually experience the following malignant disease cycle: “hospitalization-improvement-discharge-rehospitalization” (3). Despite recent advances in the diagnosis and treatment of HF, patient mortality remains high (4, 5), suggesting that many risk factors for HF remain unexplored.

Intestinal microorganisms are involved in the occurrence and progression of many diseases (6). Recent studies have revealed the associations between human gut microbes and HF (7, 8). As one of the metabolic products in intestinal microorganisms, trimethylamine N-oxide (TMAO) has been demonstrated to promote the development of HF and is an important risk factor for patients with HF (9). Schuett et al. (10) followed more than 4,000 patients with HF for 9.7 years; and they reported that after excluding factors, such as body weight, smoking, hypertension, diabetes, and atrial fibrillation, high levels of TMAO are associated with all-cause mortality in patients with HF. TMAO aggravates HF by damaging vascular endothelial function, affecting mitochondrial metabolism, and promoting myocardial fibrosis (11, 12). Owing to the limited sample size, further evaluation is still needed.

Therefore, the present systematic review and meta-analysis aimed to explore the prognostic value of TMAO in HF. To our knowledge, this is the first study to include data from around the world describing the association between TMAO and HF.

## Methods

### Study Registration

This meta-analysis was performed in accordance with the recommendations of the Preferred Reporting Items for Systematic Reviews (PRISMA) statement (13). The review protocol was registered at PROSPERO as CRD42021267208.

### Search Strategy

We searched the following 4 English electronic databases and 3 Chinese literature databases for studies published from inception to June 1, 2021: PubMed, Excerpta Medica Database (EMBASE), Cochrane Library, Web of Science, Wanfang database, SINOMED, VIP database, and China National Knowledge Infrastructure (CNKI). In addition, there was no restriction for language. Taking PubMed as an example, the details of the search strategy are shown in Table 1. This work was completed by two independent reviewers (ZF and JC), and in cases where they disagreed, a third individual (LX) was consulted. Moreover, manual retrieval was performed on Baidu Academic, Google Academic, books, impurities, and conference materials to obtain all the materials related to this study as comprehensively as possible. We also screened the references of the included papers, full texts, and bibliographies of all potential articles, including relevant reviews and meta-analyses, to identify additional eligible studies.

**Table 1:** Search strategy in the PubMed database.

**Number**	**Search terms**
#1	Heart failure [Mesh]
#2	(Cardiac Failure [Title/Abstract]) OR (Heart Decompensation [Title/Abstract])) OR (Decompensation, Heart [Title/Abstract])) OR (Heart Failure, Right-Sided [Title/Abstract])) OR (Heart Failure, Right Sided [Title/Abstract])) OR (Right-Sided Heart Failure [Title/Abstract])) OR (Right Sided Heart Failure [Title/Abstract])) OR (Myocardial Failure [Title/Abstract])) OR (Congestive Heart Failure [Title/Abstract])) OR (Heart Failure, Congestive [Title/Abstract])) OR (Heart Failure, Left-Sided [Title/Abstract])) OR (Heart Failure, Left Sided [Title/Abstract])) OR (Left-Sided Heart Failure [Title/Abstract])) OR (Left Sided Heart Failure [Title/Abstract])
#3	#1OR#2
#4	(“trimethylamine N-oxide” [Mesh])
#5	(trimethyloxamine [Title/Abstract]) OR (trimethylammonium oxide [Title/Abstract]) OR (TMAO [Title/Abstract]) OR (trimethylamine oxide [Title/Abstract])
#6	#4OR#5
#7	#3AND#6

### Inclusion and Exclusion Criteria

To prevent bias, the inclusion criteria were prespecified as follows: (a) the subjects were patients with HF; (b) prospective cohort; (c) major adverse cardiac events (MACEs), including cardiovascular mortality, MI, cardiovascular hospitalization or revascularization, in addition to all-cause mortality were reported; and (d) hazard ratio (HR)/relative risk (RR) and 95% CI were reported.

Studies were excluded if any of the following criteria were observed: (a) duplications; (b) lack of data on TMAO levels and heart failure; or (c) case reports, animal trials, review articles, systematic reviews, meta-analyses, commentaries, editorials, or meeting abstracts.

### Data Extraction

All data were independently extracted by two reviewers (DL and JL), and disagreements were resolved by discussion. If necessary, a third author (XC) was involved. Study information of the included studies was recorded, including the first author, publication year, participant characteristics, sample size, region, circulating TMAO concentration, duration of follow-up, adjusted risk factors, and outcome assessment.

### Quality Assessment

Two authors (XC and LX) independently assessed the methodological quality of the included studies. The Newcastle-Ottawa quality assessment scale (NOS) was used to evaluate the risk of bias based on study group selection, group comparability, and ascertainment of exposure or outcome (14). The maximum score of this scale is 9 points, and studies with a score ≥7 are rated as high quality (15).

### Data Synthesis and Statistical Analysis

The data were analyzed with Stata (version 13.0) (16). RRs and 95% CIs were used to estimate the combined effects. The overall effect was calculated by a *Z*-test, and *P* < 0.05 (2-tailed) was defined as statistically significant. Potential heterogeneity was evaluated by Cochran Q and *I*^2^ statistics (17). A fixed-effect model was employed when heterogeneity was low (*P* ≥ 0.05, *I*^2^ ≤ 50%) (18). However, when high heterogeneity occurred (*P* < 0.05, *I*^2^ > 50%), we further analyzed its potential sources from the following three aspects: clinical heterogeneity, methodological heterogeneity, and statistical heterogeneity. We evaluated clinical heterogeneity first. If there was evident clinical heterogeneity, subgroup analysis was performed. If clinical heterogeneity was evident and subgroup analysis could not be conducted, descriptive analysis was only used. After excluding clinical and methodological heterogeneity, statistical heterogeneity was considered, and a random-effect model was used (19). A funnel chart, Egger's, and Begg's test were constructed to evaluate publication bias (19). Publication bias will be adjusted by the method of trim and filling.

We performed sensitivity analyses to evaluate reliable results. The methods included changing the type of analysis methods (random-effects model or fixed-effects model), eliminating each of the included studies one by one, and then combining the effect quantities.

### Ethics

Because patient privacy was not involved in the present study, ethical approval was not needed.

## Results

### Literature Search and Screening

**Figure 1:**
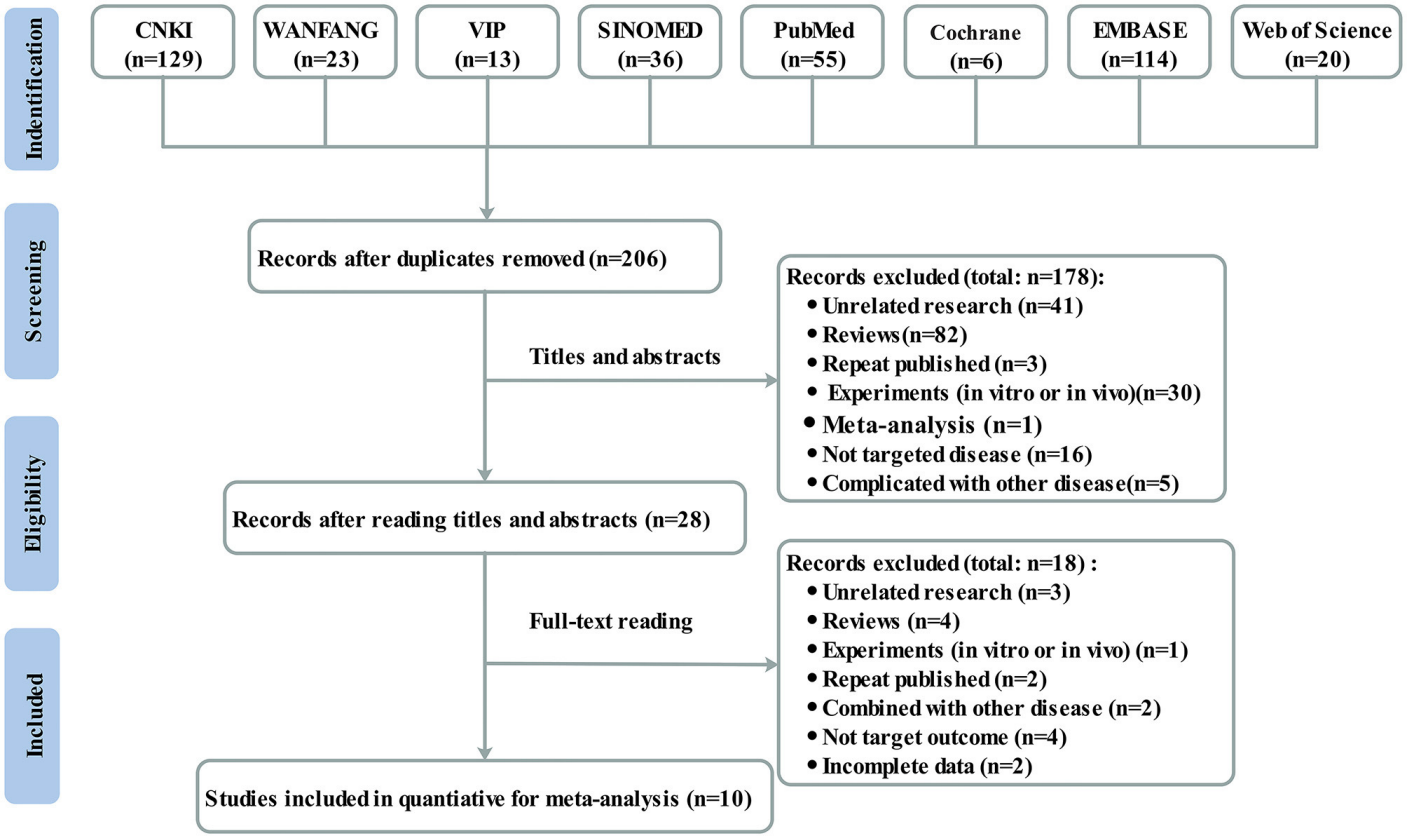
Flowchart of study selection.

The search of 8 databases identified 396 articles for further evaluation (129 from CNKI, 23 from WANFANG, 13 from VIP, 36 from SINOMED, 55 from PubMed, 6 from the Cochrane Library, 114 from Embase, and 20 from the Web of Science), of which 190 were removed after review due to duplicate records. After reading the titles and abstracts, 178 were excluded for various reasons. Finally, only 10 studies (10, 20–28) met the inclusion criteria after screening full texts. Figure 1 shows the detailed process of the study selection process.

### Study Characteristics and Quality Assessment

Ten articles (12 studies) involving 13,425 participants from 2014 to 2021 were included in the present study. Most trials were performed in Europe (3 in the UK, 1 in Norway, 1 in Germany, and 1 in other); however, trials were also performed in the United States (2 trials) and Asia (2 in China). Only three studies (23, 24, 28) included patients with acute HF; all others included patients with chronic HF. Three studies were multicenter, and the others were single-center. The follow-up duration ranged from 1 to 9.7 years with a median follow-up of 3.4 years. The average age of the patients ranged from 57 to 78 years old. Most trials had more males than females. Baseline TMAO levels were reported in all included studies. All the studies reported data on MACEs or all-cause mortality. All observational studies were adjusted for multiple potential confounding factors, and more than half of the studies were rated as “good” quality. The characteristics of the included studies are listed in Table 2.

**Table 2:** Characteristics of the selected studies and quality assessment.

**References**	**Location**	**HF type**	**Scale**	**Follow-up, years**	**Participants (male, %)**	**Age, years**	**TMAO level (umol/L)**	**Outcome**	**Adjusted risk factors**	**Study quality**
Tang et al. (20)	USA	Chronic	Single center	5	720 (59)	66 ± 10	5.0 (3.0, 8.5)	All-cause mortality	Yes	8
Tang et al. (21)	USA	Chronic	Single center	5	112 (75)	57 ± 14	5.8 (3.6, 12.1)	All-cause mortality	Yes	8
Trøseid et al. (22)	Norway	Chronic	Single center	5.2	155 (83)	57 ± 11	NA	All-cause mortality	Yes	7
Suzuki and Heaney (23)	UK	Acute	Single center	1	972 (61)	78 (69–84)	5.6 (3.4, 10.5)	All-cause mortality	Yes	6
Schuett et al. (10)	Germany	Chronic	Single center	9.7	2,490 (NA)	63 ± 10	4.73 (3.4, 6.82)/4.73 (3.22, 6.85)	All-cause mortality	Yes	6
Liu (24)	China	Acute	Single center	1	64 (67)	70 ± 14	6.56 ± 11.83	All-cause mortality	Yes	6
Suzuki et al. (25)	European	Chronic	Multicenter	1	2,234 (74)	70 (61–78)	5.9 (3.6, 10.8)	All-cause mortality; MACE	Yes	8
Suzuki et al. (25)	European	Chronic	Multicenter	2	2,234 (74)	70 (61–78)	5.9 (3.6, 10.8)	All-cause mortality; MACE	Yes	8
Suzuki et al. (25)	European	Chronic	Multicenter	3	2,234 (74)	70 (61–78)	5.9 (3.6, 10.8)	All-cause mortality; MACE	Yes	8
Salzano et al. (26)	UK	Chronic	Single center	5	196 (49)	73 (67–78)	7.0 (4.2, 12.5)	All-cause mortality	Yes	6
Zhou et al. (27)	China	Chronic	Multicenter	1.8	1,208 (689)	73 (64–80)	4.5 (2.83, 7.92)	All-cause mortality, MACE	Yes	7
Israr et al. (28)	UK	Acute	Single center	1	806 (61)	78 (69–84)	10.2 (5.8, 18.7)	All-cause mortality; MACE	Yes	6

*USA, the United States; UK, United Kingdom*.

### Primary Outcome: The Association Between TMAO Level and MACEs in Patients With HF

**Figure 2:**
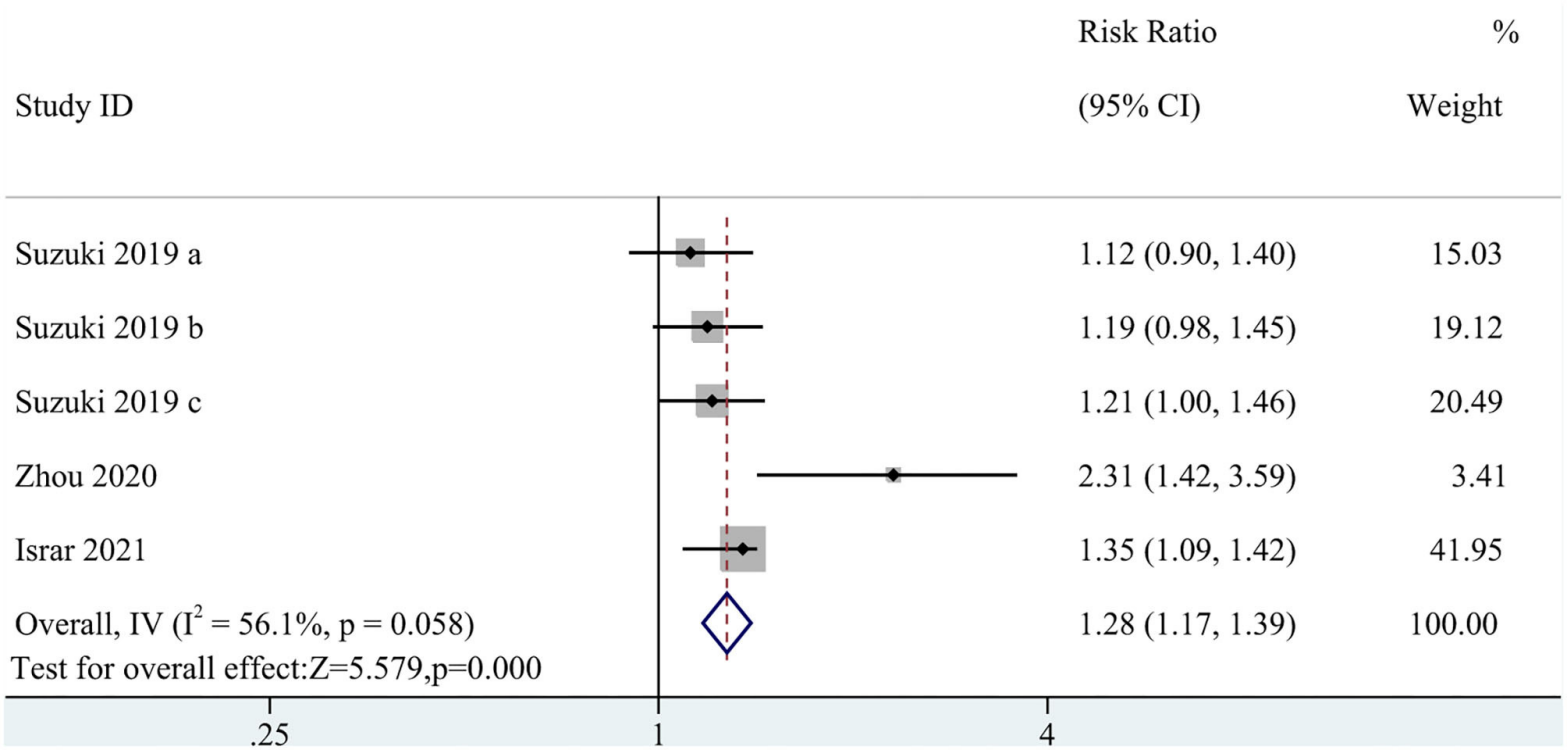
Forest plot for the association between trimethylamine N-oxide (TMAO) level and major adverse cardiovascular events (MACEs).

**Figure 3:**
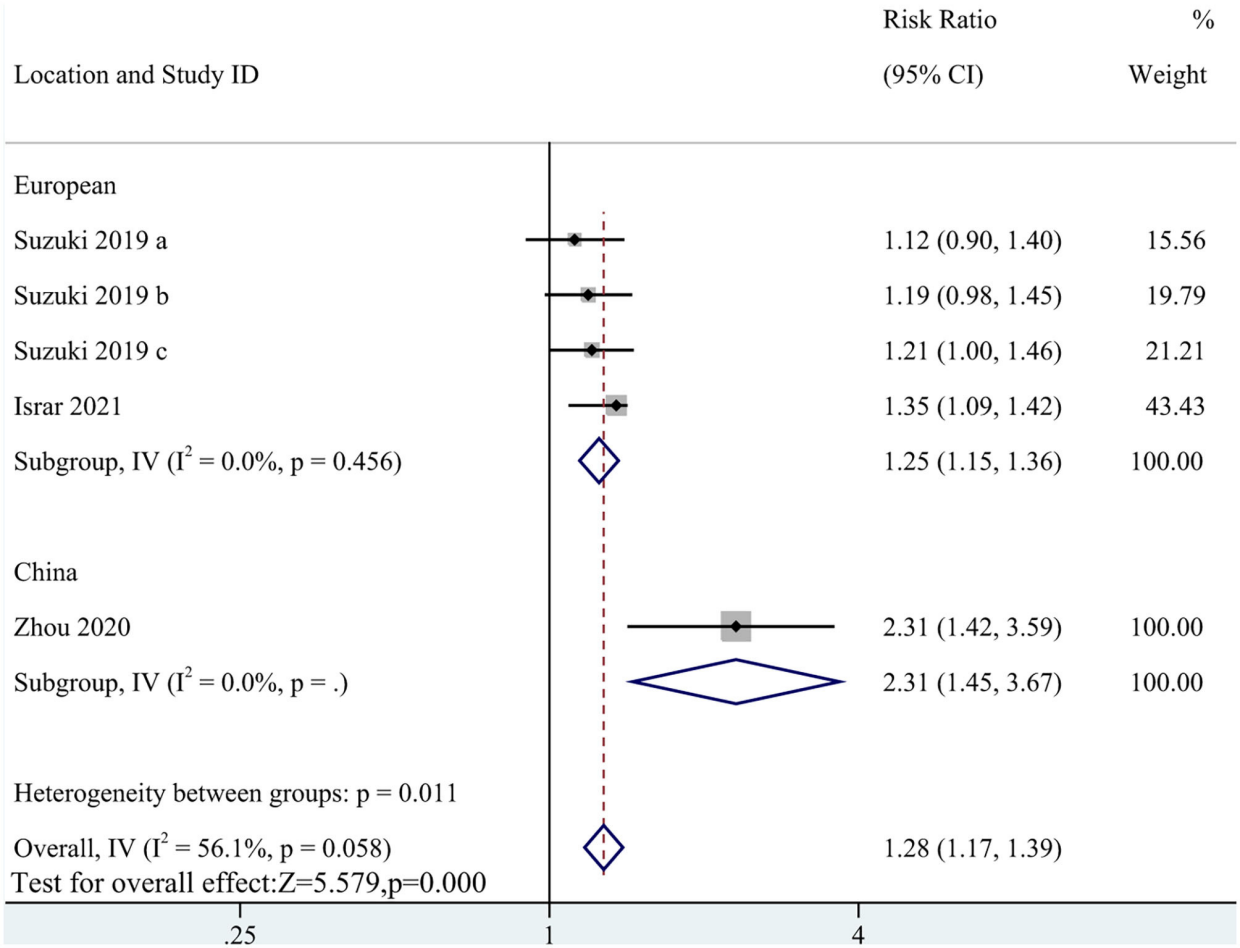
Subgroup analysis for the association between TMAO level and MACEs.

Five cohort studies with a total of 8,716 participants reported an association of MACEs with TMAO levels (RR: 1.28; 95% CI: 1.17, 1.39; *P* < 0.0001; *I*^2^= 56.1%; *P*-heterogeneity = 0.058; random-effects model; Figure 2). This result indicated that a high circulating TMAO concentration was associated with a greater risk of MACEs in patients with HF. Due to the high heterogeneity, subgroup analysis was implemented. The subgroup analysis did not affect the results (Figure 3).

### Secondary Outcome: The Association Between TMAO Level and All-Cause Mortality in Patients With HF

**Figure 4:**
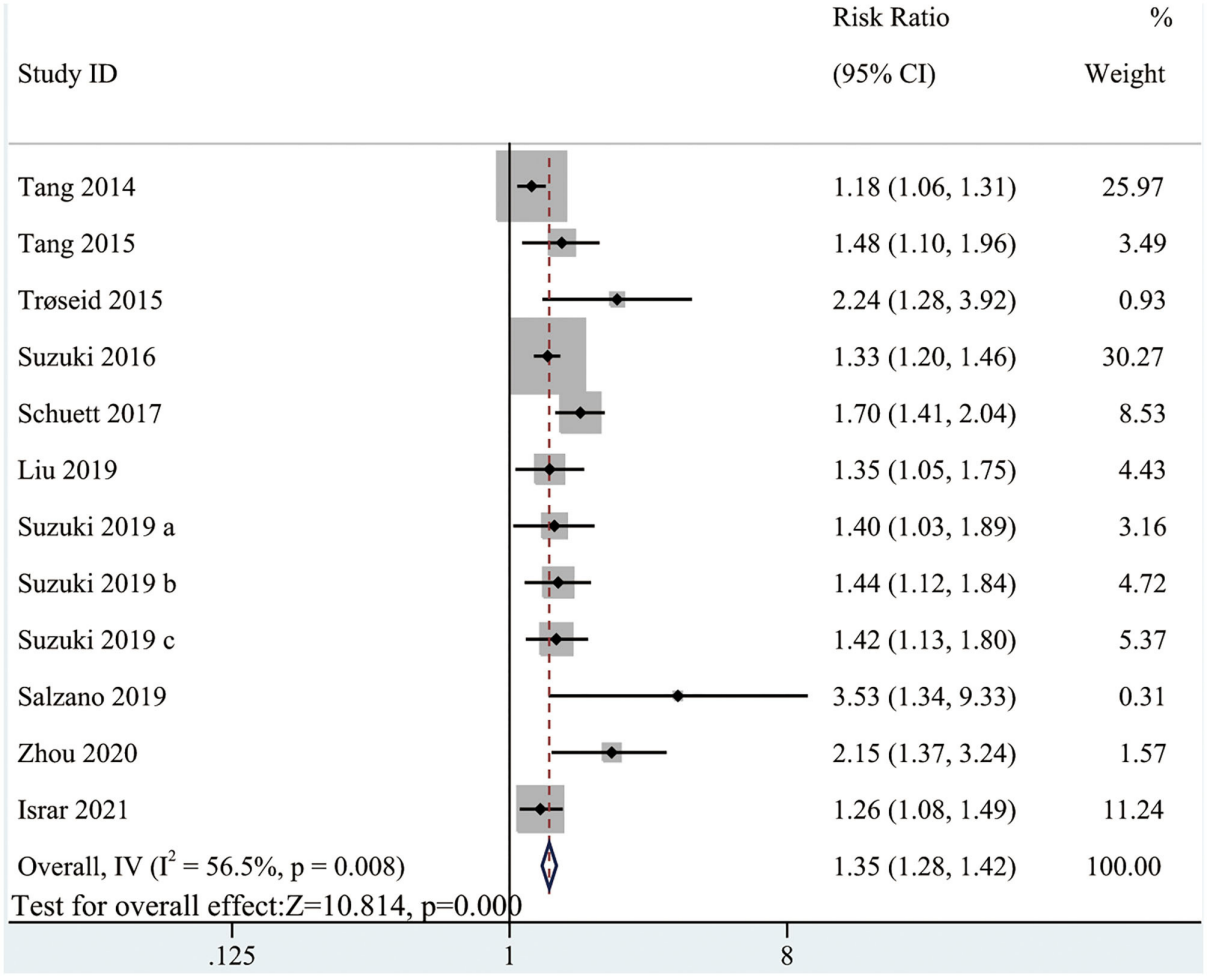
Forest plot for the association between TMAO level and all-cause mortality.

The pooled analysis comparing the all-cause mortality in HF patients with high and low circulating TMAO concentrations involved all included studies (RR: 1.35; 95% CI: 1.28, 1.42; *P* < 0.0001; *I*^2^ = 56.5%; *P*-heterogeneity = 0.008; random-effects model; Figure 4). The results showed that the risk of all-cause mortality was greater with higher circulating TMAO concentrations among patients with HF. Considering significant heterogeneity, we performed subgroup analysis based on study characteristics to explore the potential sources of heterogeneity (Table 3). Seven items, including study scale, location, HF type, sample size, age, follow up, and study quality, were included in the subgroup analysis to evaluate the impact on heterogeneity. In general, the results were not influenced by these factors, indicating that the meta-analysis result was stable.

**Table 3:** Subgroup analysis for the association between TMAO level and all-cause mortality according to study characteristics.

**Subgroups**	**Studies, *n***	**Effects model**	**Over effect**		**Heterogeneity**	
			**RR (95% CI)**	***P*** **value**	***I*** **^2^, %**	***P*** **value**
All	12	Random	1.35 (1.28, 1.42)	<0.0001	56.5	0.008
Scale
Single-center	8	Fixed	1.32 (1.25, 1.40)	<0.0001	64.8	0.006
Multi-center	4	Random	1.49 (1.29, 1.71)	<0.0001	5.8	0.364
Location
USA	2	Random	1.21 (1.19, 1.34)	<0.0001	52	0.149
European	8	Fixed	1.40 (1.31, 1.49)	<0.0001	47	0.067
Asia	2	Random	1.53 (1.22, 1.90)	<0.0001	69.8	0.069
HF type
Chronic	9	Random	1.34 (1.28, 1.48)	<0.0001	67	0.002
Acute	3	Fixed	1.31 (1.21, 1.42)	<0.0001	0	0.834
Participants
<900	6	Random	1.26 (1.16, 1.36)	<0.0001	55.9	0.045
≥900	6	Fixed	1.43 (1.32, 1.54)	<0.0001	44.1	0.112
Age
<70	5	Random	1.33 (1.22, 1.44)	<0.0001	74.3	0.004
≥70	7	Fixed	1.36 (1.27, 1.46)	<0.0001	36.8	0.148
Follow up
<5	7	Fixed	1.35 (1.26, 1.45)	<0.0001	0	0.449
≥5	5	Random	1.33 (1.22, 1.46)	<0.0001	79.4	0.001
Study quality
Good	8	Random	1.32 (1.24, 1.40)	<0.0001	51.0	0.046
Fair	4	Random	1.43 (1.29, 1.67)	<0.0001	67.6	0.026

### Publication Bias Analysis

**Figure 5:**
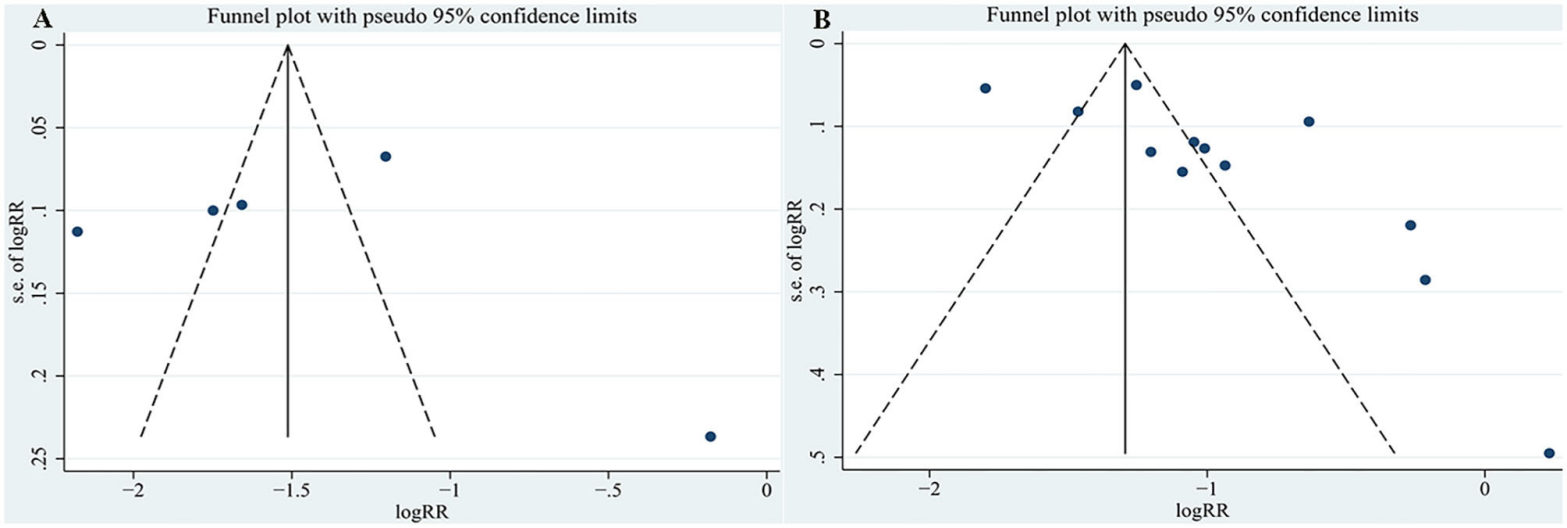
Funnel plot for publication bias. **(A)** Funnel plot for publication bias of the association between TMAO level and MACEs; **(B)** Funnel plot for publication bias of the association between TMAO level and all-cause mortality.

**Figure 6:**
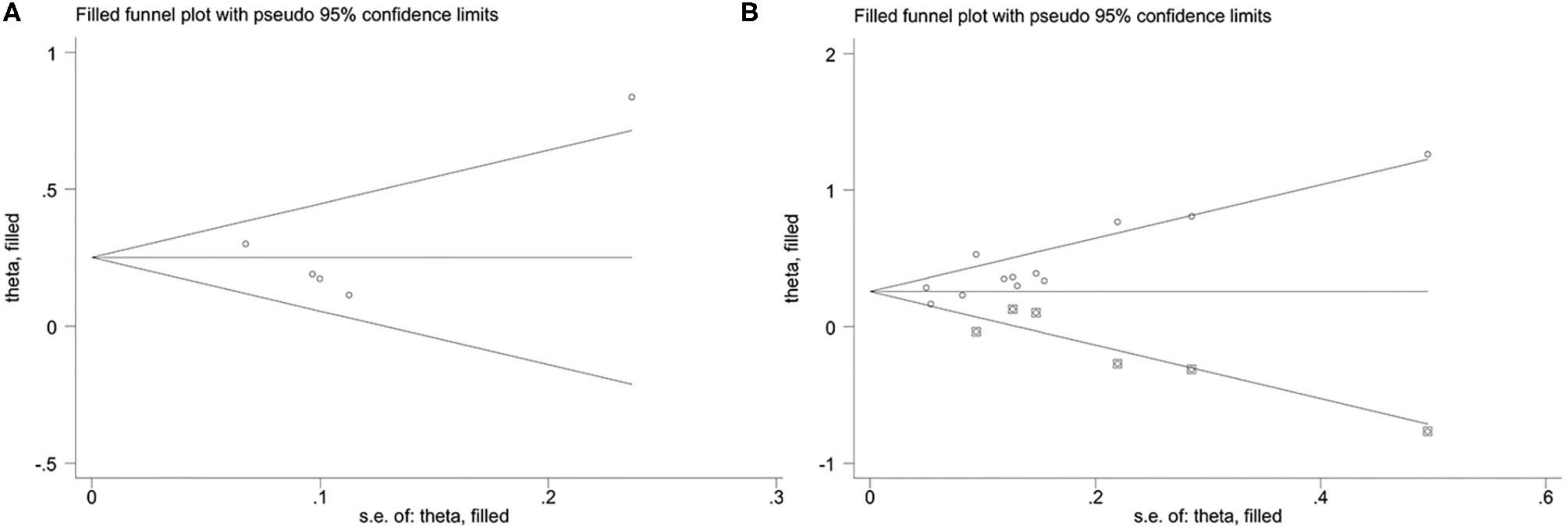
Trim and fill method on MACEs **(A)** and all-cause mortality **(B)**.

For the relationship between TMAO and MACEs in the present study, the funnel plot appeared asymmetric (Figure 5A), but further Egger's and Begg's tests demonstrated that there was no publication bias (*P* = 0.922 for Egger's test and *P* = 0.462 for Begg's test). In addition, the funnel plot and Egger's test all indicated potential publication bias for the relationship between TMAO and all-cause mortality (*P* = 0.017 for Egger's test; Figure 5B). Although publication bias existed, the results of trim-and-fill method suggested that the meta-analysis results were robust (RR: 0.251; 95% CI: 0.11, 0.392; *P* < 0.001 for MACEs; RR: 0.257; 95% CI: 0.153, 0.36; *P* < 0.001 for all-cause mortality; Figure 6).

### Sensitivity Analysis

**Figure 7:**
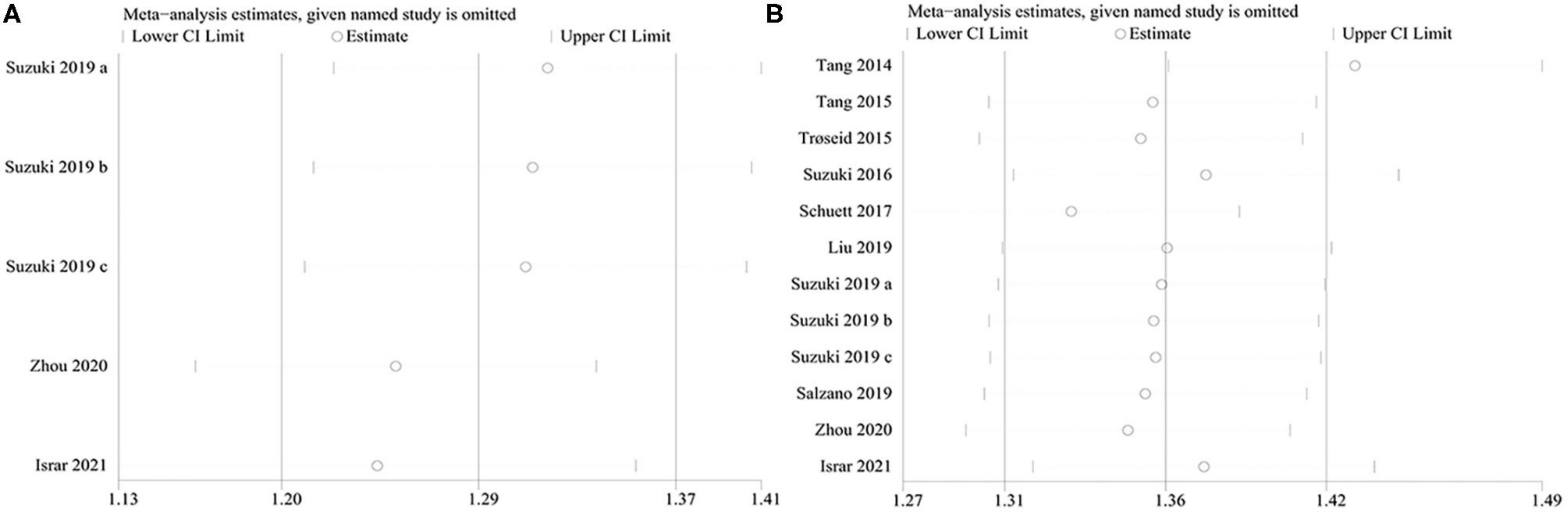
The sensitivity analysis of the study. **(A)** The sensitivity analysis of the association between TMAO level and MACEs; **(B)** The sensitivity analysis of the association between TMAO level and all-cause mortality.

Sensitivity analyses were performed to assess whether the results of this meta-analysis were stable. The results showed that no obvious effect was found after deleting the studies one by one, which suggested that the study results were credible (Figure 7).

## Discussion

At present, there are few reported meta-analyses on the relationship between TMAO and the poor prognosis of HF; the included studies are mainly from Europe and the United States, and there is a lack of data on Asian HF populations (29). With this background, we performed the first systematic review and meta-analysis involving studies from around the world to explore whether high levels of TMAO are related to the poor prognosis of HF patients. These 10 articles were published from 2014 to 2021, which reflected recent results about the role of circulating TMAO levels in predicting the poor prognosis of patients with HF. This systematic review and meta-analysis provided relatively reliable evidence that elevated TMAO is related to MACEs and all-cause mortality in patients with HF.

**Figure 8:**
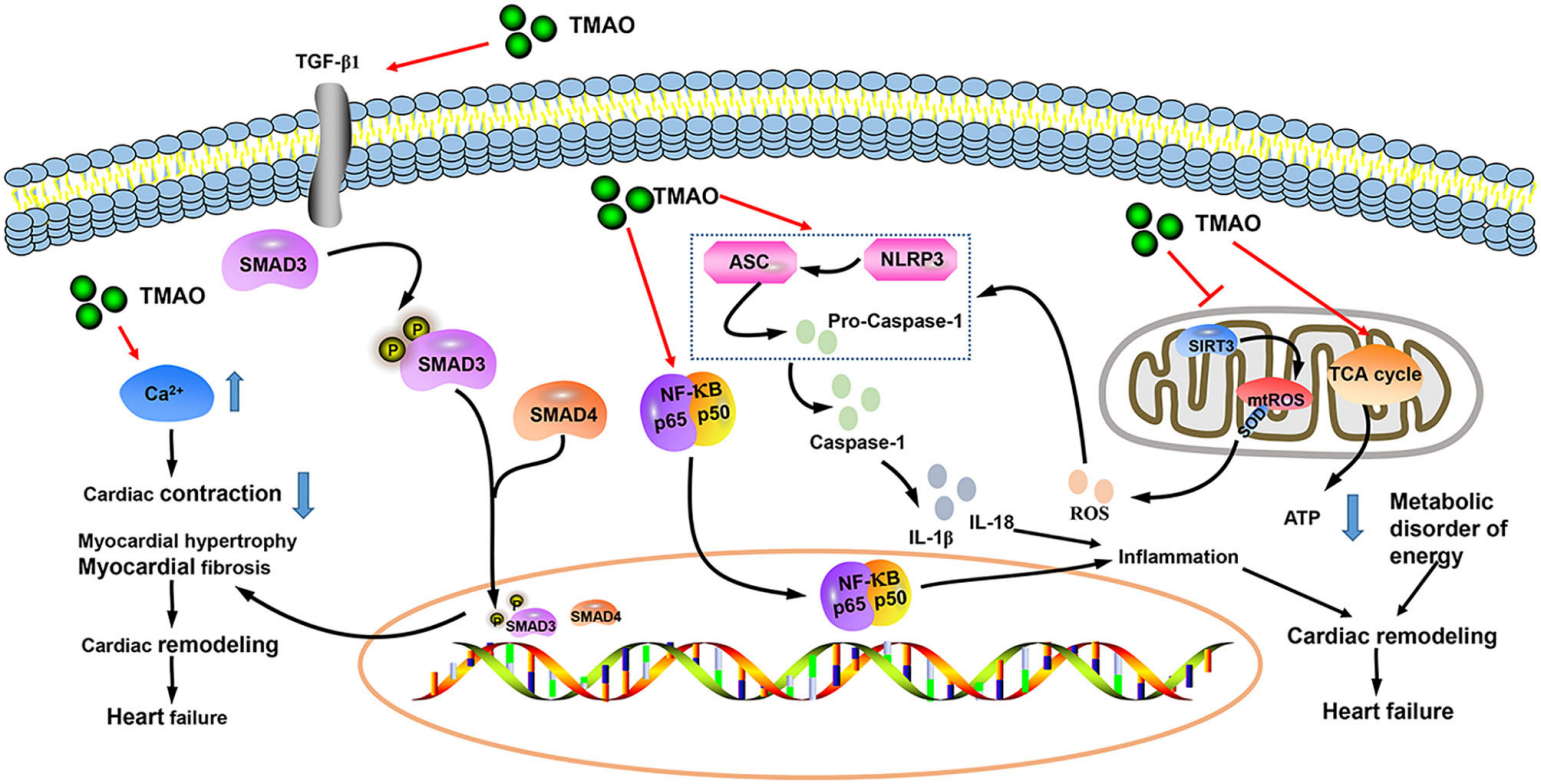
Mechanism of TMAO exacerbating heart failure (HF) progression.

Trimethylamine N-oxide (TMAO) is a small odorless molecule (molecular mass of 75.11 g/mol) (30). Dietary choline, betaine, and L-carnitine are metabolized by intestinal microorganisms to generate trimethylamine (TMA), which is further converted to TMAO by hepatic flavin monooxygenase (FMO) (31, 32). Although TMAO can maintain normal physiological activities of the human body, high concentrations of TMAO may cause various cardiovascular diseases (33). Animal studies have shown that both choline supplementation (a precursor to TMAO production) and direct dietary TMAO supplementation increase the level of TMAO, exacerbating myocardial fibrosis and worsening cardiac function (34). Chen et al. (35) proposed that inhibitors of TMA formation reduce circulating TMAO levels in obese mice induced by Western diets, thereby preventing subsequent cardiac dysfunction. TMAO induces HF *via* multiple pathological mechanisms, including inflammation, mitochondrial dysfunction, oxygen-free radical production, and myocardial fibrosis (Figure 8) (36–40). There are many factors that affect the level of TMAO in the body, including diet, intestinal microbiota, sex, age, kidney function, weight, and drugs (31, 32, 41, 42). In the original studies included in this article, 7 were corrected for age, 5 were corrected for renal function, and 4 were corrected for sex and weight. At the same time, we conducted subgroup analyses of age, scale, location, HF type (chronic or acute), sample size, follow-up, and study quality to explore the potential sources of heterogeneity. Sensitivity analysis was also implemented. Fortunately, subgroup analyses and sensitivity analyses did not change the overall results. We obtained the same result as Li et al. (29), but our original studies were distributed in Europe, the United States, and Asia, which made up for the deficiency of their study. However, it is worth mentioning that we still cannot rule out the influence of diet, gut microbiota, and drugs on TMAO. Red meat, eggs, and sea fish are common sources of TMAO in the diet (31, 41, 43), thereby reminding nutritionists to pay attention to the nutritional benefits of these foods while not ignoring the adverse metabolites (such as TMAO) associated with these foods when formulating nutritional prescriptions for patients with cardiovascular disease.

The rapidly increasing population of patients with HF imposes a heavy social and economic burden. Our results encourage the exploration of interventions to reduce TMAO. At present, methods to reduce TMAO mainly remain in the stage of animal experiments, including 3,3-dimethyl-1-butanol, choline analogs (IMC and FMC), and betaine aldehyde (44–46). The method used to reduce TMAO in clinical is microbial-driven therapy, however, a meta-analysis showed the opposite conclusion (47).

Existing studies have revealed the key role of TMAO in HF. TMAO may participate in the occurrence and development of HF through a variety of mechanisms. However, related scientific research has just started, and only limited studies have revealed the connection between TMAO and HF. There are questions about the relationship between TMAO and HF. First, due to the lack of long-term monitoring of circulating TMAO concentration in patients before HF occurs, it is still difficult to determine whether a high level of TMAO is the triggering factor of HF. The above results provided preliminary evidence that TMAO predicts the poor prognosis of patients with HF. Our findings originated from clinical data, adding new direct evidence, which largely confirms the reliability of the association between high concentrations of TMAO and poor prognosis of HF. Second, TMAO reduction interventions remain in the laboratory stage, and there is no effective treatment in the clinic. Finally, the results of the dose-response relationship provided in the meta-analysis better present the relationship between different dose levels of exposure factors and outcomes, making the results more reliable. However, due to the lack of studies on the relationship between different concentrations of TMAO and the prognosis of HF, the dose-response relationship between TMAO and the poor prognosis of HF remain unknown.

In the future, a large-scale prospective cohort should be further developed to explore the causal relationship between TMAO and HF, starting before the occurrence of HF. At the same time, the relationship between different concentrations of TMAO and the poor prognosis of patients with HF should be discussed. Prospective multicenter randomized controlled trials should be performed to further advance the understanding of TMAO from the laboratory to the clinic to treat patients with HF.

## Strengths and Limitations

The present study clarified that TMAO is related to the poor prognosis of patients with HF. Moreover, more than 60 percent of the original studies were of high quality. The present study includes research from around the world, thereby allowing the research results to be widely promoted and overcoming the deficiencies of existing research to a certain extent. The detailed description of baseline characteristics also allowed identification of the source of heterogeneity.

However, the present study had several limitations. First, although we established rigorous inclusion criteria, some results indicated publication bias. Second, there was a lack of studies on the relationship between different concentrations of TMAO and MACE or all-cause death in HF, preventing a dose-response meta-analysis. Finally, we were unable to explore all the variables that influence TMAO levels, such as diet, environment, and medications.

## Conclusion

The present study demonstrated that TMAO may be a risk factor for poor prognosis in patients with HF, but the underlying mechanism requires further investigation. Developing therapies that directly target TMAO may constitute a novel approach for preventing and treating HF.

## Data Availability Statement

The raw data supporting the conclusions of this article will be made available by the authors, without undue reservation.

## Author Contributions

XL and YL: conceptualization and writing original draft. YW and QL: project administration. ZF, JC, and DL: data management and data analysis. JL, XC, and LX: methodology and software application. XL, YL, and QL: writing review and editing and funding acquisition. All authors read and approved the final manuscript, critically reviewed the literature, and contributed to drafting the manuscript.

## Funding

This work was supported by the Capital's Funds for Health Improvement and Research (Grant No. 2020-2-4201), National Natural Science Foundation of China (81973622), and Beijing University of Chinese Medicine 1166 Development Program for Junior Scientists (No. 030903010331).

## Conflict of Interest

The authors declare that the research was conducted in the absence of any commercial or financial relationships that could be construed as a potential conflict of interest.

## Publisher's Note

All claims expressed in this article are solely those of the authors and do not necessarily represent those of their affiliated organizations, or those of the publisher, the editors and the reviewers. Any product that may be evaluated in this article, or claim that may be made by its manufacturer, is not guaranteed or endorsed by the publisher.

## References

[1] Tromp J Bamadhaj S Cleland JGF Angermann CE Dahlstrom U Ouwerkerk W . Post-discharge prognosis of patients admitted to hospital for heart failure by world region, and national level of income and income disparity (REPORT-HF): a cohort study. Lancet Glob Health. (2020) 8:e411–22. 10.1016/S2214-109X(20)30004-8 32087174

[2] Collaborators GDaIIaP. Global, regional, and national incidence, prevalence, and years lived with disability for 354 diseases and injuries for 195 countries and territories, 1990-2017: a systematic analysis for the Global Burden of Disease Study 2017. Lancet. (2018) 392:1789–858. 10.1016/S0140-6736(18)32279-7 30496104 PMC6227754

[3] Savarese G Sattar N Januzzi J Verma S Lund LH Fitchett D . Empagliflozin is associated with a lower risk of post-acute heart failure rehospitalization and mortality. Circulation. (2019) 139:1458–60. 10.1161/CIRCULATIONAHA.118.038339 30855996

[4] Rossignol P Hernandez AF Solomon SD Zannad F. Heart failure drug treatment. Lancet. (2019) 393:1034–44. 10.1016/S0140-6736(18)31808-7 30860029

[5] McDonagh TA Metra M. 2021 ESC Guidelines for the diagnosis and treatment of acute and chronic heart failure. Eur Heart J. (2021). 42:3599–726. 10.1093/eurheartj/ehab368 34447992

[6] Sata Y Marques FZ Kaye DM. The emerging role of gut dysbiosis in cardio-metabolic risk factors for heart failure. Curr Hypertens Rep. (2020) 22:38. 10.1007/s11906-020-01046-0 32385705

[7] Tang WHW Li DY Hazen SL. Dietary metabolism, the gut microbiome, and heart failure. Nat Rev Cardiol. (2019) 16:137–54. 10.1038/s41569-018-0108-7 30410105 PMC6377322

[8] Madan S Mehra MR. Gut dysbiosis and heart failure: navigating the universe within. Eur J Heart Fail. (2020) 22:629–37. 10.1002/ejhf.1792 32168550

[9] Zhang Y Wang Y Ke B Du J. TMAO: how gut microbiota contributes to heart failure. Transl Res. (2021) 228:109–25. 10.1016/j.trsl.2020.08.007 32841736

[10] Schuett K Kleber ME Scharnagl H Lorkowski S März W Niessner A . Trimethylamine-N-oxide and heart failure with reduced versus preserved ejection fraction. J Am Coll Cardiol. (2017) 70:3202–4. 10.1016/j.jacc.2017.10.064 29268932

[11] Ma G Pan B Chen Y Guo C Zhao M Zheng L . Trimethylamine N-oxide in atherogenesis: impairing endothelial self-repair capacity and enhancing monocyte adhesion. Biosci Rep. (2017) 37:BSR20160244. 10.1042/BSR20160244 28153917 PMC5333780

[12] Chen ML Zhu XH Ran L Lang HD Yi L Mi MT. Trimethylamine-N-Oxide induces vascular inflammation by activating the NLRP3 inflammasome through the SIRT3-SOD2-mtROS signaling pathway. J Am Heart Assoc. (2017) 6:e002238. 10.1161/JAHA.117.006347 28871042 PMC5634285

[13] Shamseer L Moher D Clarke M Ghersi D Liberati A Petticrew M . Preferred reporting items for systematic review and meta-analysis protocols (PRISMA-P) 2015: elaboration and explanation. BMJ. (2015) 350:g7647. 10.1136/bmj.g7647 25555855

[14] Stang A. Critical evaluation of the Newcastle-Ottawa scale for the assessment of the quality of nonrandomized studies in meta-analyses. Eur J Epidemiol. (2010) 25:603–5. 10.1007/s10654-010-9491-z 20652370

[15] Zhou QP Li XJ. C-reactive protein to albumin ratio in colorectal cancer: a meta-analysis of prognostic value. Dose Response. (2019) 17:1559325819889814. 10.1177/1559325819889814 31798355 PMC6868585

[16] Higgins JP Altman DG Gøtzsche PC Jüni P Moher D Oxman AD . The Cochrane Collaboration's tool for assessing risk of bias in randomised trials. BMJ. (2011) 343:d5928. 10.1136/bmj.d5928 22008217 PMC3196245

[17] Higgins JP Thompson SG Deeks JJ Altman DG. Measuring inconsistency in meta-analyses. BMJ. (2003) 327:557–60. 10.1136/bmj.327.7414.557 12958120 PMC192859

[18] Lau J Ioannidis JP Schmid CH. Quantitative synthesis in systematic reviews. Ann Intern Med. (1997) 127:820–6. 10.7326/0003-4819-127-9-199711010-00008 9382404

[19] Borenstein M Hedges LV Higgins JP Rothstein HR. A basic introduction to fixed-effect and random-effects models for meta-analysis. Res Synth Methods. (2010) 1:97–111. 10.1002/jrsm.12 26061376

[20] Tang WH Wang Z Fan Y Levison B Hazen JE Donahue LM . Prognostic value of elevated levels of intestinal microbe-generated metabolite trimethylamine-N-oxide in patients with heart failure: refining the gut hypothesis. J Am Coll Cardiol. (2014) 64:1908–14. 10.1016/j.jacc.2014.02.617 25444145 PMC4254529

[21] Tang WH Wang Z Shrestha K Borowski AG Wu Y Troughton RW . Intestinal microbiota-dependent phosphatidylcholine metabolites, diastolic dysfunction, and adverse clinical outcomes in chronic systolic heart failure. J Card Fail. (2015) 21:91–6. 10.1016/j.cardfail.2014.11.006 25459686 PMC4312712

[22] Trøseid M Ueland T Hov JR Svardal A Gregersen I Dahl CP . Microbiota-dependent metabolite trimethylamine-N-oxide is associated with disease severity and survival of patients with chronic heart failure. J Intern Med. (2015) 277:717–26. 10.1111/joim.12328 25382824

[23] Suzuki T Heaney LM. Trimethylamine N-oxide and prognosis in acute heart failure. Heart. (2016) 102:841–8. 10.1136/heartjnl-2015-308826 26869641

[24] Liu YQH. The Relationship Between Plasma Trimethylamine Oxide Level and Poor Prognosis in Patients With Acute Heart Failure. Hunan: Nanhua University (2019).

[25] Suzuki T Yazaki Y Voors AA Jones DJL Chan DCS Anker SD . Association with outcomes and response to treatment of trimethylamine N-oxide in heart failure: results from BIOSTAT-CHF. Eur J Heart Fail. (2019) 21:877–86. 10.1002/ejhf.1338 30370976

[26] Salzano A Israr MZ Yazaki Y Heaney LM Kanagala P Singh A . Combined use of trimethylamine N-oxide with BNP for risk stratification in heart failure with preserved ejection fraction: findings from the DIAMONDHFpEF study. Eur J Prev Cardiol. (2020) 27:2159–62. 10.1177/2047487319870355 31412713

[27] Zhou X Jin M Liu L Yu Z Lu X Zhang H. Trimethylamine N-oxide and cardiovascular outcomes in patients with chronic heart failure after myocardial infarction. ESC Heart Fail. (2020) 7:188–93. 10.1002/ehf2.12552 31960610 PMC7083407

[28] Israr MZ Bernieh D Salzano A Cassambai S Yazaki Y Heaney LM . Association of gut-related metabolites with outcome in acute heart failure. Am Heart J. (2021) 234:71–80. 10.1016/j.ahj.2021.01.006 33454370

[29] Li W Huang A Zhu H Liu X Huang X Huang Y . Gut microbiota-derived trimethylamine N-oxide is associated with poor prognosis in patients with heart failure. Med J Aust. (2020) 213:374–9. 10.5694/mja2.50781 32959366

[30] Bennett BJ de Aguiar Vallim TQ Wang Z Shih DM Meng Y Gregory J . Trimethylamine-N-oxide, a metabolite associated with atherosclerosis, exhibits complex genetic and dietary regulation. Cell Metab. (2013) 17:49–60. 10.1016/j.cmet.2012.12.011 23312283 PMC3771112

[31] Jonsson AL Bäckhed F. Role of gut microbiota in atherosclerosis. Nat Rev Cardiol. (2017) 14:79–87. 10.1038/nrcardio.2016.183 27905479

[32] Ma J Li H. The role of gut microbiota in atherosclerosis and hypertension. Front Pharmacol. (2018) 9:1082. 10.3389/fphar.2018.01082 30319417 PMC6167910

[33] Zhu Y Li Q Jiang H. Gut microbiota in atherosclerosis: focus on trimethylamine N-oxide. Apmis. (2020) 128:353–66. 10.1111/apm.13038 32108960 PMC7318354

[34] Organ CL Otsuka H Bhushan S Wang Z Bradley J Trivedi R . Choline diet and its gut microbe-derived metabolite, trimethylamine N-Oxide, exacerbate pressure overload-induced heart failure. Circ Heart Fail. (2016) 9:e002314. 10.1161/CIRCHEARTFAILURE.115.002314 26699388 PMC4943035

[35] Chen K Zheng X Feng M Li D Zhang H. Gut microbiota-dependent metabolite trimethylamine N-Oxide contributes to cardiac dysfunction in western diet-induced obese mice. Front Physiol. (2017) 8:139. 10.3389/fphys.2017.00139 28377725 PMC5359299

[36] Li Z Wu Z Yan J. Gut microbe-derived metabolite trimethylamine N-oxide induces cardiac hypertrophy and fibrosis. Lab Invest. (2019) 99:346–57. 10.1038/s41374-018-0091-y 30068915

[37] Sun X Jiao X Ma Y Liu Y Zhang L He Y . Trimethylamine N-oxide induces inflammation and endothelial dysfunction in human umbilical vein endothelial cells via activating ROS-TXNIP-NLRP3 inflammasome. Biochem Biophys Res Commun. (2016) 481:63–70. 10.1016/j.bbrc.2016.11.017 27833015

[38] Seldin MM Meng Y Qi H Zhu W Wang Z Hazen SL . Trimethylamine N-Oxide promotes vascular inflammation through signaling of Mitogen-Activated Protein Kinase and Nuclear Factor-κB. J Am Heart Assoc. (2016) 5:e002767. 10.1161/JAHA.115.002767 26903003 PMC4802459

[39] ML C XH Z L R HD L L Y MT M. Trimethylamine-N-Oxide induces vascular inflammation by activating the NLRP3 inflammasome through the SIRT3-SOD2-mtROS signaling pathway. J Am Heart Assoc. (2017). 6:e002238. 10.1161/JAHA.117.002238 28871042 PMC5634285

[40] Savi M Bocchi L. Trimethylamine-N-Oxide (TMAO)-Induced impairment of cardiomyocyte function and the protective role of Urolithin B-Glucuronide. Molecules. (2018) 23:549. 10.3390/molecules23030549 29494535 PMC6017162

[41] Janeiro MH Ramírez MJ Milagro FI. Implication of Trimethylamine N-Oxide (TMAO) in disease: potential biomarker or new therapeutic target. Nutrients. (2018) 10:1398. 10.3390/nu10101398 30275434 PMC6213249

[42] Yang S Li X Yang F Zhao R Pan X Liang J . Gut microbiota-dependent marker TMAO in promoting cardiovascular disease: inflammation mechanism, clinical prognostic, and potential as a therapeutic target. Front Pharmacol. (2019) 10:1360. 10.3389/fphar.2019.01360 31803054 PMC6877687

[43] Cho CE Taesuwan S Malysheva OV Bender E Tulchinsky NF Yan J . Trimethylamine-N-oxide (TMAO) response to animal source foods varies among healthy young men and is influenced by their gut microbiota composition: a randomized controlled trial. Mol Nutr Food Res. (2017) 61:1600324. 10.1002/mnfr.201600324 27377678

[44] Wang Z Roberts AB Buffa JA Levison BS Zhu W Org E . Non-lethal inhibition of gut microbial trimethylamine production for the treatment of atherosclerosis. Cell. (2015) 163:1585–95. 10.1016/j.cell.2015.11.055 26687352 PMC4871610

[45] Roberts AB Gu X Buffa JA Hurd AG Wang Z Zhu W . Development of a gut microbe-targeted nonlethal therapeutic to inhibit thrombosis potential. Nat Med. (2018) 24:1407–17. 10.1038/s41591-018-0128-1 30082863 PMC6129214

[46] Orman M Bodea S Funk MA. Structure-guided identification of a small molecule that inhibits anaerobic choline metabolism by human gut bacteria. J Am Chem Soc. (2019) 141:33–7. 10.1021/jacs.8b04883 30557011 PMC6475491

[47] Miao L Du J Chen Z Shi D Qu H. Effects of microbiota-driven therapy on circulating Trimethylamine-N-Oxide Metabolism: a systematic review and meta-analysis. Front Cardiovasc Med. (2021) 8:710567. 10.3389/fcvm.2021.710567 34552967 PMC8450403

